# The behaviour of T2* and T2 relaxation time in extrinsic foot muscles under continuous exercise: A prospective analysis during extended running

**DOI:** 10.1371/journal.pone.0264066

**Published:** 2022-02-17

**Authors:** Charlotte Zaeske, Gert-Peter Brueggemann, Steffen Willwacher, Daniela Maehlich, David Maintz, Grischa Bratke

**Affiliations:** 1 Institute for Diagnostic and Interventional Radiology, Faculty of Medicine and University Hospital Cologne, Cologne, Germany; 2 Institute of Biomechanics and Orthopaedics, German Sport University, Cologne, Germany; McLean Hospital, UNITED STATES

## Abstract

**Objectives:**

Previous studies on T2* and T2 relaxation time of the muscles have shown that exercise leads to an initial increase, presumably representing different intramuscular physiological processes such as increase in intracellular volume or blood oxygenation level dependent effects with a subsequent decrease after cessation of exercise. Their behaviour during prolonged exercise is still unknown but could provide important information for example about the pathophysiology of overuse injuries. The aim of this study was to evaluate the temporal course of T2* and T2 relaxation time in extrinsic foot muscles during prolonged exercise and determine the optimal mapping technique.

**Methods:**

Ten participants had to run a total of 75 minutes at their individual highest possible running speed, with interleaved MR scans at baseline and after 2.5, 5, 10, 15, 45 and 75 minutes. The examined extrinsic foot muscles were manually segmented, and relaxation time were analysed regarding its respective time course.

**Results:**

T2* and T2 relaxation time showed an initial increase, followed by a plateau phase between 2.5 and 15 minutes and a subsequent decrease. For the T2* relaxation time, this pattern was also apparent, but less pronounced, with more muscles not reaching significance (p<0.05) when comparing different time points.

**Conclusions:**

T2* and T2 relaxation time showed a similar course with an initial rapid increase, a plateau phase and a subsequent decrease under prolonged exercise. Moderate but long-term muscular activity appears to have a weaker effect on T2* relaxation time than on T2 relaxation time.

## Introduction

Conventional MRI is an important tool in the diagnosis of muscular injuries. However, in advanced diagnostics, e. g. in the detection of microtrauma, in the monitoring of muscular recovery and in the imaging of exercise induced effects, these techniques are brought to their limits. Different new techniques, such as quantitative MRI (including for example the measurement of relaxation times, diffusion tensor imaging or MR spectroscopy) show promising results in imaging these processes [1–5] and bear the potential of becoming an important tool in further studies of exercise physiology. In contrast to conventional "qualitative" visual inspection of images, quantitative imaging aims to obtain measurable physical or chemical data that can be compared between different tissue regions and between different individuals [6].

In terms of the measurement of T2* and T2 relaxation times, as one possible application of quantitative MRI, both of the parameters represent time constants and refer to the time it takes for the signal to decrease to 37% of the original value during transverse relaxation of magnetisation. While T2 relaxation time refers to the “true” transverse relaxation, caused by atomic and molecular interactions, T2* relaxation time describes the “observed” transverse relaxation, caused by the effects of “true” transverse relaxation as well as magnetic field inhomogeneities [7]. Consequently, T2* relaxation times are expected to be less or equal to the correlating T2 relaxation times [7–9]. These parameters can be calculated by acquisition of multiple images with different echo times (TE), using gradient echo sequences to calculate T2* relaxation times and spin echo sequences to calculate T2 relaxation times [7]. With this approach, the sensitivity of MRI to biophysical properties of tissue is sought to be converted into quantitative image information [9].

Previous studies on exercise induced effects on the muscle have shown that muscular activation leads to an increase of the T2* and T2 relaxation times [4, 10, 11], with the possibility of determining patterns of spatial distribution as well as quantifying the intensity of muscle activation [12–14]. In consideration of the underlying physiology, it is currently assumed that the increase in T2 relaxation time is caused by an increase of the muscular water content due to osmotically induced increase in extra- and intracellular volume and intracellular acidification by products of metabolism, such as lactate, phosphate and sodium [12, 14–16]. The physiological behaviour of T2* relaxation time is less well investigated so far. In contrast to the T2 relaxation time, a relationship to the blood oxygen level dependent (BOLD) contrast is suspected here, which results from the microvascular ratio of oxyhemoglobin to deoxyhemoglobin and reflects tissue oxygenation as well as oxygen extraction at the microvascular level [7, 13].

To date, existing literature provides direct pre- and post-exercise comparisons of T2* and T2 relaxation times and detailed reports on the first 15-minutes of exercise, leaving it unclear how prolonged exercise affects the T2* and T2-relaxation time [17, 18]. However, information on signal behaviour during prolonged exercise could provide insight into long-term physiological processes that may have clinical implications regarding the causes and avoidance strategies for overuse injuries. Compared to other forms of aerobic exercise such as swimming, walking or cycling, especially running is associated with a higher risk of overuse injury with the highest proportion of injuries occurring distal to the knee [19]. Many of these injuries are related to muscle dysfunction or fatigue [20] and include pathologies such as tendinitis, chronic compartment syndrome of the tibialis anterior muscle, shin splints or stress fractures [20].

The purpose of this study was to investigate the temporal behaviour of T2* and T2 relaxation time in all the extrinsic foot muscles during a 75-minute running training session and determine the optimal mapping technique.

## Material and methods

This prospective study was approved by the local institutional review board (Review board of the Medical Faculty of the University of Cologne, registration number 16–375) and registered in the German Clinical Trials Registry (DRKS00011152). According to the Declaration of Helsinki, a written informed consent was obtained from all study participants. Participants and image data were acquired between December 2016 and August 2018. Ten participants (6 males and 4 females) were recruited from the German Sport University Cologne for the study. Inclusion criteria were recreational activity at least twice a week and an age between 18 and 40 years. This upper age limit was set because it is known that T2 relaxation time of fast-twitch muscles increases with age, presumably mainly due to the increased extracellular space [21]. Minimizing the age range was intended to prevent any influence of age on the results. Exclusion criteria were neurological or cardiovascular diseases or sports-related injuries at the time of the study. Participants with neurological and cardiovascular diseases were not included to avoid (serious) adverse events related to the participant’s health (e. g. exercise-induced cardiac arrhythmias, cardiac decompensation) and relevant confounding factors related to exercise capacity (e. g. failure to reach maximum speed due to cardiovascular reasons or neuromuscular disabilities) or muscular structure (e. g. in neuromuscular disorders). None of the participants wore orthotics while running. All participants were instructed not to exercise on the day of the study.

### Exercise protocol

All the participants were equipped with neutral running shoes with a homogeneous density ethyl vinyl acetate midsole. A treadmill (F85, Sole Fitness, Neu-Ulm, Germany) was placed directly in front of the MRI scanner room. To avoid additional physical effort or differences in timing, the distance between MRI scanner and treadmill was limited to less than 5 steps. The MRI scans were performed immediately after running to avoid possible regeneration effects.

**Fig 1:**
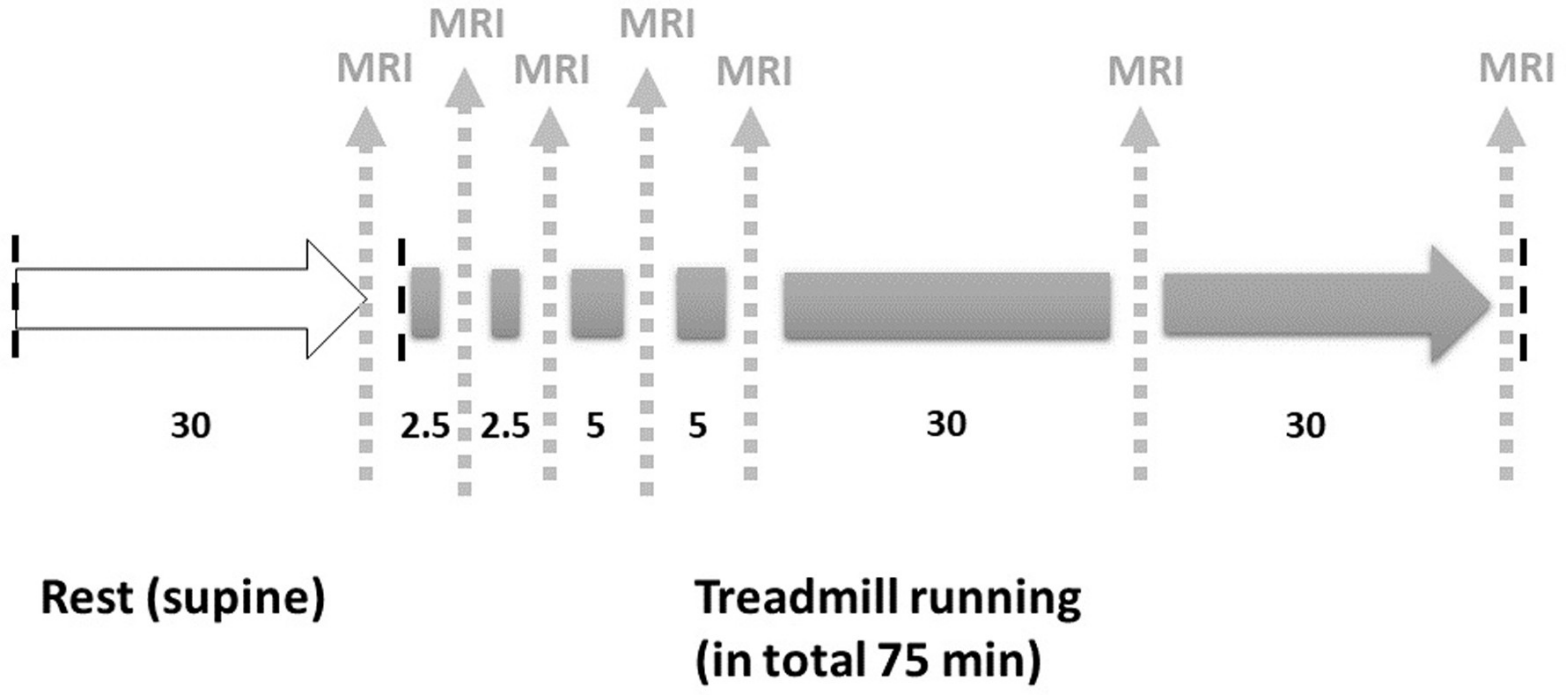
Schematic diagram of the scanning and running procedure. Vertical black broken lines indicate (from left to right) the start of the protocol, the onset of running and the end of running. Vertical grey broken lines indicate the interleaved MRI-Scans.

During a short initial test procedure consisting of a few seconds of running on the treadmill, each participant chose the highest possible running speed that could be maintained for 75 minutes according to their experience. Two of the participants completed this before the exercise (prior to their rest period), the remaining participants completed the measurement on a different day. This initial test procedure resulted in running speeds from 8.5 to 12.0 km/h. With regard to the results of Fisher et. al, the participants first had to undergo a thirty-minute rest period on the MR table outside the MR scanner to neutralize any muscle activation before the experiment [17]. Here, the participants were positioned on a MR table in a relaxed, supine position with slightly bent knees (about 20°) with the support the base of the knee coil. Subsequently, the participants could be transported into the scanner without any further muscle activity. After this rest period the pre-run scan followed. During the run, MRI scans of the right calf were performed after 2.5, 5, 10, 15, 45 and 75 minutes (Fig 1). The treadmill was not stopped or slowed down for participants to get on and off for MRI scans to ensure a constant running speed. The respective time periods were chosen in order to achieve a high temporal resolution of the initial changes in muscle activity with an expected, rapid increase in T2* and T2 relaxation time, with consecutive longer bouts to map the following time course.

The start time of each scan began less than one minute after the end of the run. The scans interrupted the running time by 3:40 minutes on average. The respective time periods concerning scan duration, running time on the treadmill and switching between scanner and treadmill were recorded with a stopwatch.

### MRI examination

All multiple-slice MRI scans were performed on a 3-T scanner (Philips Ingenia 3.0 T, Philips Healthcare, Best, The Netherlands) using a 16-channel high-resolution transmitting and receiving knee coil. A custom-made MR-compatible board was fixed on the MR table to position the foot at the same spot after each bout to ensure consistency between all scans of an individual participant. For each participant, the coil base was left fixed on the MR table while the right lower leg was positioned centrally in the coil base. The initial scan included T1-weighted sequences in coronal and axial orientation for optimal anatomical planning. Subsequently, T2* and T2 maps were acquired as described below, starting with the T2* maps in each case.

A Carr-Meibom-Purcell sequence similar to that described by Willwacher et al [22] (differing in the number of echoes and echo times) and comparable to Schuermans et al [23] was used to generate T2 maps after acquisition of 16 echoes in the range of 10 to 160 ms (10, 20, 30, 40, 50, 60, 70, 80, 90, 100, 110, 120, 130, 140, 150, 160) with a repetition time of TR = 2000 ms (flip angle: 90 degrees, SENSE factor: 1.7, voxel size: 1.5 x 1.5 x 5 mm^3^, slice thickness 5 mm, acquisition time 1:00 min). T2* maps were generated after acquiring 12 echoes in the range of 5 to 390 ms (5, 8.2, 11.4, 14.6, 17.8, 21, 24.2, 27.4, 30.6, 33.8, 37, 40.2) with a repetition time of TR 490 ms (flip angle: 25 degrees, SENSE factor 1.7, voxel size 1.5 x 1.5 x 5 mm^3^, slice thickness 5 mm, acquisition time: 0:40 min). T2* and T2 relaxation time was calculated by the scanner based on the exponential decay of the signal intensity. As a mono-exponential fit was used to estimate the T2 value, differences between water and fat T2 relaxation times, when present in the same voxel, were not modelled in the current work. The estimated T2 values represent an effective T2 comprised of the proton density weighted fractions of water and fat in each voxel. B1 inhomogeneities were not corrected, however, as a knee coil with a comparable small diameter was used for signal transmission, the B1 variation across the region of interest can be considered small. For both of the maps, ten slices with a 15 mm gap were acquired, which resulted in an identical FOV with a craniocaudal length of 185 mm, starting from the fibula head, and transversal dimensions of 150 x 197 mm^2^.

### Data analysis

A total of six slices of the MRI scans were used for the analysis. The first and last two slices of each scan were excluded, due to a poor signal-to-noise ratio or field inhomogeneities that occurred in some scans. For each slice and at each point in time, the external foot muscles were manually segmented by tracing the outline of the muscle bellies, excluding intramuscular vessels and intramuscular fat, for all acquired six slices for each time point. Segmentation was performed on the first echo, as it provided the best contrast for the anatomical structures, and then copied to the calculated T2*-map and T2-map respectively. An example of the segmentation of the T2* and T2 maps is included in the supplement.

Segmentation contained the medial (MG) and lateral gastrocnemius (LG), soleus (SO), peroneus longus (PER), tibialis posterior (TP), tibialis anterior (TA) and extensor digitorum longus (EDL). Vessels and intramuscular fat were excluded. The segmentation was conducted by a radiologist specialized in musculoskeletal imaging (GB, seven years of experience) using HOROS viewer 3.3.2 (The Horos Project, Annapolis, MD, USA).

To calculate the average T2 relaxation time (and T2* relaxation time respectively) of each muscle for a given time, the following formula was used, based on previous work of Ploutz-Snyder et al. [24]:

$$T2\;relaxation\;time_{m}{}_{uscle}=\sum_{slice1}^{slice6}relaxation\;time_{\;slice\;x}\times \frac{CSA_{slice\;x}}{CSA_{all\;six\;slices}}.$$

For each muscle and time point, the relaxation time of each slice was multiplied with the cross-sectional area (CSA) in relation to the total cross-sectional area of the muscle (ACSA). The results for each slice were then summed up. This calculation ensures that the relaxation time for each slice is included according to its percentage of total muscle volume. The muscle volumes of the participants have previously been used by Willwacher et al [22] and Bratke et al [25].

### Statistical analysis

Data of T2* and T2 relaxation times were tested for normal distribution using the Shapiro-wilk test. For parametric data, a one-way variance analysis (ANOVA) for repeated measurements was performed with a Geisser-Greenhouse correction, followed by a post-hoc Tukey test with correction for multiple comparisons. For nonparametric data, Friedman test for nonparametric, matched data was performed with a consecutively performed Dunn’s test with correction for multiple comparisons. Based on the preliminary results, an isolated comparison of the 15 min time point with the 45 min time point was added for the T2* and T2 relaxation times of the different muscles using a nonparametric Wilcoxon matched-pairs signed rank test.

Effect sizes were reported as η^2^ for one-way analyses of variance (with the underlying definition of values <0.06 as small effect size, from 0.06–0.14 as moderate effect size and >0.14 as large effect size) and as Kendall’s W-value for the Friedman test (with underlying definition of values from 0.1–0.29 as small effect size, from 0.3–0.49 as moderate effect size and >0.5 as large effect size), referred to as w. Effect size refers to the magnitude of difference between group means, respectively the strength of a statistical effect, and serves to illustrate the practical relevance of statistically significant results [26, 27]. In contrary to the probability of error, reported by p-value of statistical significance, a measure of effect size is expected to be useful for comparing results from different tests or samples of different sizes [27].

The correlations of the variables were analysed by calculation of correlation matrices using nonparametric Spearman correlation coefficients. Variables included the difference in T2* and T2 relaxation times from baseline to minute 2.5, labelled as "increase", and each patient’s height, weight, speed, age and gender.

All data are reported as mean ± standard deviation (SD). A p-value of <0.05 was considered statistically significant. Statistical analyses were performed using GraphPad Prism Version 8.4.1 (GraphPad Software Inc., San Diego, California, USA).

## Results

Data on average demographic and physiological characteristics of the recruited participants are outlined in Table 1. Recreational activities performed by participants included field hockey, soccer, basketball, athletics and triathlon, at least twice a week.

**Table 1:** Demographic and physiologic characteristics of the subjects.

Age, years	28.70 ± 2.83
BMI, kg/m^2^	22.91 ± 2.10
Height, cm	175,80 ± 5.46
Velocity, km/h	9.34 ±1.54
Gender	6 males, 4 females

Data given as mean ± standard deviation for age, BMI and velocity. Total number of subjects tested: n = 10.

The actual times of the individual running sessions largely corresponded to the targeted times with 02:31,53 ± 00:01,86 for the 2.5 minute running segments, 05:01,09 ± 00:03,61 for the 5 minute running segments and 30:01,82 ± 00:03,61 for the 30 minute running segments, respectively. The total time of interruption of the running sessions was 03:43,02 ± 00:36,03, consisting of the scan time (02:13,94 ± 00:11,17) and the transitions times between scanner and treadmill (01:03,28 ± 00:23,04) and vice versa (00:42,24 ± 00:07,96).

### Segmented muscles

Testing for normal distribution revealed a normal distribution for the T2 relaxation times of MG, TP and PER and a normal distribution for the T2* relaxation times of MG, SOl, TP, PER, EDL and TA. Regarding the conducted one-way ANOVA and Friedman tests for T2 relaxation time, there was a significant main effect for all muscles (MG, LG, SOL, PER, EDL, TA: all p<0.0001, TP: p = 0.0002). With regard to T2* relaxation time, the main effect was significant for almost all muscles (MG, LG: <0.0001, PER: p = 0.0027, EDL: p = 0.0343, TA: p = 0.0019), with the exception of SOL (p = 0.0555) and TP (p = 0.4958). Effect sizes for T2 were as follows: MG: 0.84 (η^2^), LG: 0.70 (w), SOL: 0.55 (w), TP: 0.55 (η^2^), PER: 0.68 (η^2^), EDL: 0.68 (w), TA: 0.79 (w), indicating a strong effect for all of the muscles. Effect sizes for T2* were as follows: MG: 0.80 (η^2^), LG: 0.78 (w), SOL: 0.24 (η^2^), TP: 0.08 (η^2^), PER: 0.42 (η^2^), EDL: 0.35 (η^2^), TA: 0.59 (η^2^), indicating a strong effect size for MG, LG, PER, EDL and TA and a moderate effect for SOL and TP.

**Fig 2:**
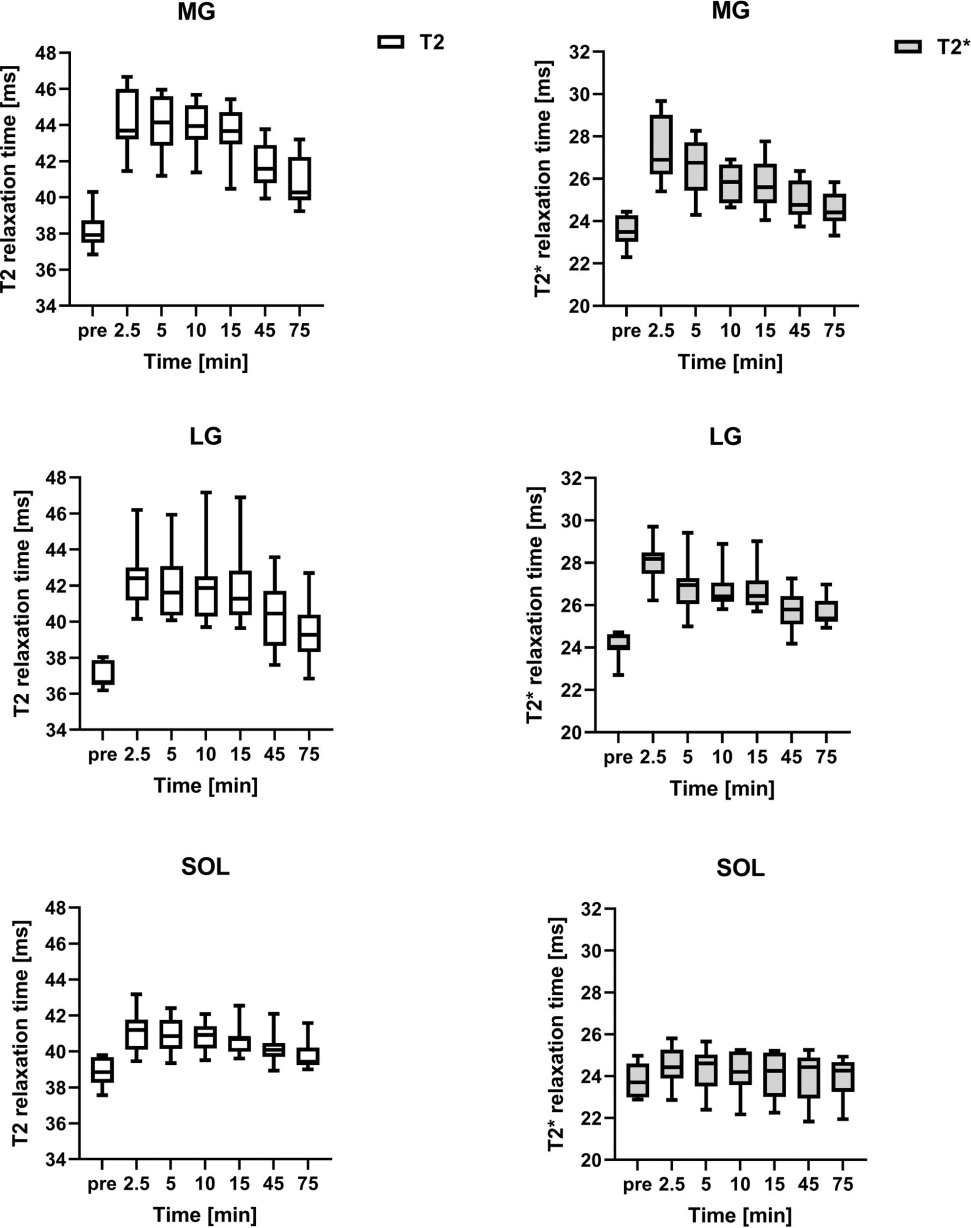
Mean T2* and T2 relaxation time of the 10 participants. Abbreviations: MG = medial gastrocnemius muscle, LG = lateral gastrocnemius muscle, SOL = soleus muscle. Note: the time intervals between the time points indicated in the x-axis are variable.

**Fig 3:**
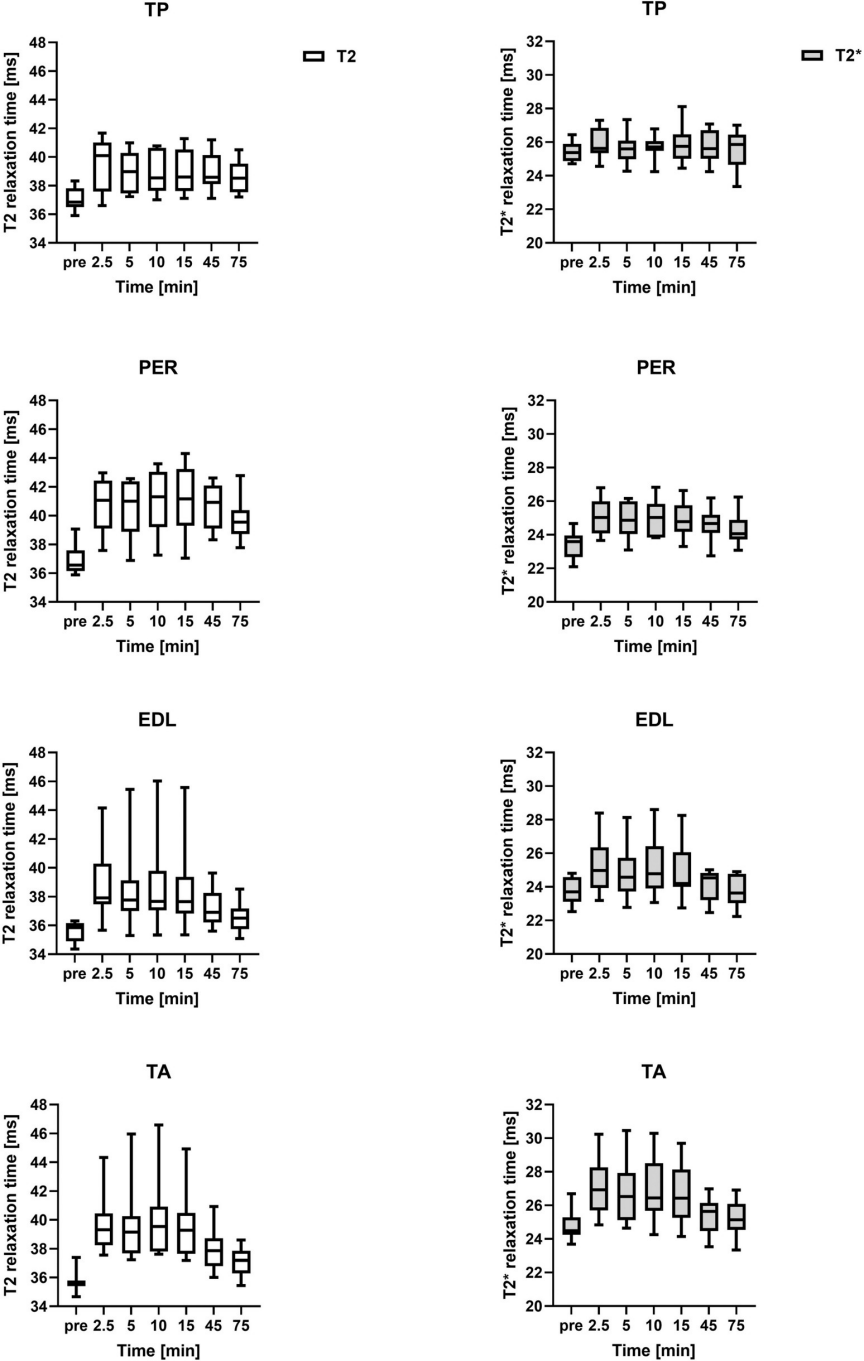
Mean T2* and T2 relaxation time of the 10 participants. Abbreviations: TP = tibialis posterior muscle, PER = peroneus longus muscle, EDL = extensor digitorum longus muscle, TA = tibialis anterior muscle. Note: the time intervals between the time points indicated in the x-axis are variable.

Regarding the temporal course, the analysis of the relaxation time showed an initial increase between baseline and 2.5 minutes for T2* and T2 relaxation time for all of the examined muscles (Figs 2 and 3 and Tables 2 and 3). The initial increase ranged from 5.90% (SOL) to 16.14% (MG) in T2 relaxation time and from 2.17% (TP) to 16.53% (LG) in T2* relaxation time. The observed increase in T2 relaxation time was statistically significant for all muscles (T2: MG, LG, EDL, TA: p <0.0001, SOL: p = 0.0011, TP: p = 0.0104, PER: p = 0.0011), while the increase in T2* relaxation time was statistically significant for almost all muscles (T2*: MG, LG: p<0.0001, PER: p = 0.0236, TA: p = 0.0094), except for SOL (p = 0.2486), TP (p = 0.4673) and EDL (p = 0.2645)

**Table 2:** Absolute and relative changes of the T2 relaxation times during prolonged running.

		Baseline	2.5 Min	5 Min	10 Min	15 Min	45 Min	75 Min
**MG**	*abs*. *[ms]*, *mean±SD*	38.15±0.98	44.31±1.72	44.04±1.54	43.96±1.29	43.59±1.45	41.78±1.30	40.85±1.39
	*rel*. *[% of BL]*	100%	116.14%	115.14%	115.21%	114.25%	109.51%	107.07%
	*Sig*. *(to BL / to 75 min)*	ns/*	*/*	*/*	*/*	*/*	*/*	*/ns
**LG**	*abs*. *[ms]*, *mean±SD*	36.95±0.71	42.48±1.79	41.98±1.85	41.95±2.11	41.90±2.10	40.41±1.94	39.39±1.64
	*rel*. *[% of BL]*	100%	114.68%	113.63%	113.55%	113.40%	109.37%	106.60%
	*Sig*. *(to BL / to 75 min)*	ns/ns	*/*	*/ns	*/*	*/*	ns/ns	ns/ns
**TP**	*abs*. *[ms]*, *mean±SD*	37.05±0.78	39.59±1.79	39.02±1.34	38.98±1.49	38.92±1.51	39.02±1.29	38.63±1.09
	*rel*. *[% of BL]*	100%	106.85%	105.32%	105.21%	105.04%	105.31%	104.27%
	*Sig*. *(to BL / to 75 min)*	ns/*	*/ns	*/ns	*/ns	*/ns	*/ns	*/ns
**SOL**	*abs*. *[ms]*, *mean±SD*	38.84±0.78	41.13±1.09	40.92±1.01	40.84±0.80	40.61±0.82	40.16±0.83	39.81±0.87
	*rel*. *[% of BL]*	100%	105.90%	105.35%	105.16%	104.57%	103.41%	102.50%
	*Sig*. *(to BL / to 75 min)*	ns/ns	*/*	*/ns	*/*	ns/ns	ns/ns	ns/ns
**PER**	*abs*. *[ms]*, *mean±SD*	36.91±1.02	40.64±1.87	40.51±2.00	41.07±2.17	41.14±2.39	40.61±1.48	39.70±1.42
	*rel*. *[% of BL]*	100%	110.11%	109.77%	111.29%	111.47%	110.03%	107.56%
	*Sig*. *(to BL / to 75 min)*	ns/*	*/ns	*/ns	*/ns	*/ns	*/ns	*/ns
**EDL**	*abs*. *[ms]*, *mean±SD*	35.60±0.67	38.93±2.75	38.53±2.95	38.74±3.15	38.55±3.06	37.23±1.31	36.55±1.01
	*rel*. *[% of BL]*	100%	109.38%	108.24%	108.84%	108.30%	104.59%	102.67%
	*Sig*. *(to BL / to 75 min)*	ns/ns	*/*	*/ns	*/*	*/ns	ns/ns	ns/ns
**TA**	*abs*. *[ms]*, *mean±SD*	35.67±0.73	39.72±2.05	39.57±2.55	39.94±2.7	39.50±2.29	37.92±1.46	37.10±1.01
	*rel*. *[% of BL]*	100%	111.35%	110.65%	111.96%	110.72%	106.30%	103.99%
	*Sig*. *(to BL / to 75 min)*	ns/ns	*/*	*/ns	*/*	*/ns	ns/ns	ns/ns

BL = Baseline, EDL = extensor digitorum longus muscle, LG = lateral gastrocnemius muscle, MG = medial gastrocnemius muscle, PER = peroneus longus muscle, SOL = soleus muscle, TA = tibialis anterior muscle, TP = tibialis posterior muscle, ns = not significant,

* = significant (p<0.05)

**Table 3:** Absolute and relative changes of the T2* relaxation times during prolonged running.

		Baseline	2.5 Min	5 Min	10 Min	15 Min	45 Min	75 Min
**MG**	*abs*. *[ms]*, *mean±SD*	23.51±0.72	27.35±1.54	26.52±1.34	25.86±0.87	25.71±1.19	24.95±0.91	24.59±0.85
	*rel*. *[% of BL]*	100%	116.32%	112.83%	110.00%	109.37%	106.15%	104.61%
	*Sig*. *(to BL / to 75 min)*	ns/*	*/*	*/*	*/*	*/ns	*/ns	*/ns
**LG**	*abs*. *[ms]*, *mean±SD*	24.06±0.58	28.03±0.93	26.89±1.16	26.72±0.89	26.70±0.98	25.77±0.88	25.63±0.67
	*rel*. *[% of BL]*	100%	116.53%	111.76%	111.08%	110.98%	107.12%	106.55%
	*Sig*. *(to BL / to 75 min)*	ns/ns	*/*	*/ns	*/ns	*/ns	ns/ns	ns/ns
**TP**	*abs*. *[ms]*, *mean±SD*	25.41±0.58	25.96±0.90	25.61±0.94	25.72±0.68	25.89±1.12	26.79±0.95	26.51±1.12
	*rel*. *[% of BL]*	100%	102.17%	100.78%	101.23%	101.88%	101.48%	100.78%
	*Sig*. *(to BL / to 75 min)*	ns/ns	ns/ns	ns/ns	ns/ns	ns/ns	ns/ns	ns/ns
**SOL**	*abs*. *[ms]*, *mean±SD*	23.81±0.82	24.47±0.85	24.29±1.01	24.19±1.01	24.10±1.03	24.04±1.11	23.93±0.98
	*rel*. *[% of BL]*	100%	102.76%	101.99%	101.59%	101.21%	100.96%	100.50%
	*Sig*. *(to BL / to 75 min)*	ns/ns	ns/ns	ns/ns	ns/ns	ns/ns	ns/ns	ns/ns
**PER**	*abs*. *[ms]*, *mean±SD*	23.40±0.80	25.05±1.10	24.88±1.12	25.07±1.07	24.93±1.05	24.61±0.93	24.31±0.96
	*rel*. *[% of BL]*	100%	107.04%	106.32%	107.15%	106.55%	105.18%	103.89%
	*Sig*. *(to BL / to 75 min)*	ns/ns	*/ns	ns/ns	*/ns	*/ns	ns/ns	ns/ns
**EDL**	*abs*. *[ms]*, *mean±SD*	23.74±0.77	25.25±1.65	24.87±1.57	25.09±1.66	24.81±1.60	24.07±0.93	23.77±0.91
	*rel*. *[% of BL]*	100%	106.38%	104.71%	105.68%	104.54%	101.43%	100.15%
	*Sig*. *(to BL / to 75 min)*	ns/ns	ns/ns	ns/ns	ns/ns	ns/ns	ns/ns	ns/ns
**TA**	*abs*. *[ms]*, *mean±SD*	24.75±0.88	27.05±1.65	26.72±1.77	26.92±1.84	26.66±1.68	25.35±1.09	25.18±1.07
	*rel*. *[% of BL]*	100%	109.33%	107.97%	108.79%	107.72%	102.46%	101.77%
	*Sig*. *(to BL / to 75 min)*	ns/ns	*/*	*/ns	*/ns	*/ns	ns/ns	ns/ns

BL = Baseline, EDL = extensor digitorum longus muscle, LG = lateral gastrocnemius muscle, MG = medial gastrocnemius muscle, PER = peroneus longus muscle, SOL = soleus muscle, TA = tibialis anterior muscle, TP = tibialis posterior muscle, ns = not significant,

* = significant (p<0.05)

In the case of the T2 relaxation time, this initial increase was followed by a plateau phase from minute 2.5 to minute 15 with a subsequent decrease in relaxation time. The plateau phase was characterised by only minor changes in relaxation time without statistical significance when comparing relaxation times from minute 2.5 to minute 15 with each other, although significant differences remained when these time points were compared to baseline. The only exception was SOL, which did not show statistical significance when comparing time point 15 to baseline. An isolated comparison of relaxation times from minute 15 to minute 45, marking the presumed end of plateau phase, revealed significant differences in MG (p = 0.002), LG (p = 0.0059), SOL (p = 0.0098), EDL (p = 0.0371) and TA (p = 0.0273).

With regard to the T2* relaxation time, this temporal course with a plateau phase from minute 2.5 to minute 15 was also apparent in LG and TA, and with some restrictions also in MG and PER (MG: significant differences when comparing timepoint 2.5 with timepoint 5, 10 and 15 / PER: no significant difference between timepoint 5 and baseline). There were no significant differences regarding the temporal course of SOL, TP and EDL. An isolated comparison of relaxation times from minute 15 to minute 45 revealed significant differences in MG (p = 0.0195), LG (p = 0.0273) and TA (p = 0.0039).

Due to the design of the study, a duration of the presumed plateau phase extending beyond minute 15 but ending before minute 45 cannot be excluded in our study, as no measurements were performed between these time points.

The subsequent decrease in relaxation time after the plateau phase beyond minute 15 resulted in increasing non-significant differences when comparing the respective time points with baseline. When comparing time point 75 with baseline, most muscles showed no significant differences in T2 relaxation time (LG, SOL, EDL, TA) and T2* relaxation time (LG, SOL, TP, EDL, TA). Exceptions regarding T2 relaxation time were MG and regarding T2* relaxation time TP and PER with preserved statistical difference.

### Correlation

The results of the correlation analysis were limited. Correlation analysis showed a significant correlation between the increase in T2 and T2* relaxation time between baseline and timepoint 2.5 Min and running speed for MG for T2 (r = -0,82, p = 0.0045) and T2* (r = -0.6525, p = 0.0460), a significant correlation between initial increase and age for SOL for T2 (r = 0.6544, p = 0.0454) and T2* (r = 0.7903, p = 0.0087) and a significant correlation between initial increase and weight for TA for T2 (r = 0.7754, p = 0.0104) and T2* (r = 0.7816, p = 0.0096).

## Discussion

The aim of our study was to observe and compare the time course of T2* and T2 relaxation time under prolonged exercise. Overall, an initial increase of T2* and T2 relaxation time was shown between the measurements at baseline and after starting the exercise, as already described in previous studies [13, 18]. This initial increase was followed by a plateau phase between minute 2.5 to 15 and a consecutive decrease of relaxation time. This is new to the previously described temporal course of the T2 and T2* relaxation time in shorter periods of exercise, where a decrease is only described after the end of the exercise [13, 18].

Similarities in the course of T2* and T2 relaxation time have already been addressed by previous studies [13, 28]. As already mentioned in the introduction, this is not surprising, as T2* and T2 are interrelated with each other. While T2 relaxation describes the “true” transverse relaxation, caused by spin-spin-interference, T2* relaxation is a combination of “true” transverse relaxation in addition to the relaxation caused by magnetic field inhomogeneities [7]. However, the attempt to correlate the measured values with physiological changes leads to different explanations. With regard to the T2* relaxation time, it is assumed that vasodilatation, as a primary response of the muscle to exercise, is initially accompanied by a a consecutively reduced oxygen extraction rate and thus a decrease in paramagnetic deoxyhemoglobin and an increase in diamagnetic oxyhemoglobin respectively [29]. While the paramagnetic deoxyhaemoglobin shortens the T2* relaxation time of the water protons, the diamagnetic oxyhaemoglobin does not influence the signal intensity of the water protons, resulting in an overall increase in the T2* relaxation time [30–32], hence presumably reflecting BOLD effects in the tissue [33–35]. The subsequent plateau phase and the following decrease in relaxation time could thus represent a short-term equilibrium between vasodilatation and oxygen extraction rate, and a consecutive decrease in T2* relaxation time due to a further increase in deoxyhemoglobin with continued exercise. Regarding T2 relaxation time, the initial increase is explained by an increase in the T2 relaxation time of intramuscular water due to an osmotically induced increase in intracellular volume and increasing intracellular acidification by metabolic products such as lactate, phosphate and sodium [3, 12]. Also, increased interstitial fluid, possibly due to increased vascular permeability due to the secretion of inflammatory substances such as histamine and prostaglandin E2, may have an effect on the initial increase of the T2 relaxation time [3]. The signal drop that occurs after the plateau phase has not yet been described. Possible physiological explanations could be a physiological adaptation due to osmolyte changes. This assumption would be supported by some studies regarding the blood lactate level, whose initial exercise-induced increase is followed by a decrease after 10–20 minutes of continuous exercise lactate [36, 37].

With regard to the differences in the time courses of the T2* and T2 relaxation time, the changes in T2* relaxation time are notably less pronounced in our study with various muscles not reaching statistical significance when comparing different time points during the running session. This stands in contrast to the results published by Varghese et al., who observed an even more pronounced initial increase of the T2* relaxation time in comparison to T2 relaxation time [13]. In the case of the gastrocnemius muscles an initial increase of approximately 4 ms (T2) and 7 ms (T2*) could be shown by Varghese et al., contrary to an increase of approximately 6 ms (T2) and 4 ms (T2*) of the medial gastrocnemius muscle and an increase of approximately 6 ms (T2) and 4 ms (T2*) of the lateral gastrocnemius muscle in our study [13]. The results of our study seem counterintuitive at first, as the higher sensitivity to susceptibilities and the BOLD effect respectively would be expected to cause stronger fluctuations of the T2* relaxation time in comparison to the T2 relaxation time. A possible explanation for these discrepancies is the different design of the studies. While the study by Varghese et al. provoked a maximum intensity of exercise, the focus of our study was on a somewhat more moderate but long-term intensity of exercise, possibly leading to more moderate changes in perfusion and oxyhaemoglobin-deoxyhaemoglobin ratio.

In total the T2* and T2 relaxation time of most muscles showed a similar temporal course. However, there are observable differences between different muscles and muscle groups regarding the individual characteristics of their response to the exercise, for example regarding the amplitude of the initial increase. A possible explanation would be a different muscle composition (e. g. regarding fibre type, mitochondrial content, glycogen capacity, oxidative phosphorylation) [38] and supposedly different function, activation and load of the individual muscle groups (e. g. eccentric vs. concentric load) [39]. In the case of the gastrocnemius muscle for example, the initial increase of T2* and T2 relaxation times was much more prominent in comparison to the other muscles. One possible explanation is, that its (predominantly) fast-twitch muscle fibres are mainly dependent on anaerobic respiration, which is associated with reduced oxygen consumption, lactate accumulation and ultimately a decrease of the pH-value [40–43]. While at the beginning of muscular activity perfusion as well as oxygen supply of all muscles increase [44], it can be assumed that the reduced oxygen consumption leads to a higher proportion of oxyhemoglobin and thus to a stronger increase in T2* times in the gastrocnemius muscle compared to muscles with a lower proportion of fast-twitch fibres. Comparing the predominantly fast-twitch gastrocnemius muscle (MG, LG) with a predominantly slow-twitch muscle with a similar function, such as the SOL [40] the initial increase of T2* and T2 relaxation time is visibly reduced in the latter, which seems to support the fact of a potential influence of fiber-type composition on the T2* and T2 relaxation time.

In addition to the structural anatomy, the functional context of each muscle should be taken into account when interpreting relaxation times. While running in general has been shown to involve predominantly eccentric contractions, the plantar flexor muscle group (including the gastrocnemius muscle) performs more concentric contractions during running, which have been shown to be associated with higher metabolic costs and greater impact on T2 relaxation times [45, 46]. This could be another possible reason for the tendency of the plantar flexor muscle group (MG, LG, SOL, TP, PER), especially the MG and LG, towards a more pronounced initial increase in T2* and T2 relaxation time in comparison to the dorsiflexor muscle group (TA, EDL).

Further comparison of the dorsiflexor and plantar flexor muscle groups also shows that the relaxation times of the dorsiflexor muscles (TA, EDL) are associated with relatively high standard deviations. This could possibly be due to the (function-related) different activation pattern in comparison to the plantar flexor muscle group [20]; however, another influence could be the differences in running speed of the volunteers. Since increased speed of gait is associated with an increase ankle dorsiflexion as well as a shift from eccentric to concentric muscle contraction in the tibialis anterior (as the main dorsiflexor) [47], this could lead to interindividual differences in T2* and T2 relaxation times. Consequently, this could be a sign that the determination of the running speed has not been conducted optimally, as explained in more detail in the limitations.

Correlation analysis revealed a significant negative correlation between the initial increase in T2* and T2 relaxation time and speed in MG, a positive correlation between the initial increase in T2* and T2 and weight in the TA and the initial increase in T2* and T2 and age in SOL. As this correlation between the initial increase and these parameters can only be shown for individual muscles, the clinical relevance here is questionable. Taking a closer look at the described correlations, a negative correlation between the initial increase in T2 relaxation time and running speed has described by our working group before, with a further description of a lower drop of T2 relaxation time during later running phases [25]. Some possible explanations would be an already altered muscle fibre composition and metabolism due to training effects or a more efficient motion pattern for better runners resulting in a reduced muscular strain [25]. A possible positive correlation between the initial increase of relaxation time and the subject’s weight can be explained theoretically as one can imagine that the acceleration of a larger weight is associated with a higher workload for the muscle with a consecutive increase in relaxation times, however, possible correlations have not been described in the literature so far. An age-related increase in T2* and T2 relaxation time has already been described in the literature [21], however related to baseline values and attributed to an increase in extracellular space, fatty infiltration and a loss in fast-twitch muscle fibres [13], thus affecting predominantly fast-twitch muscles [40]. Since the aforementioned effects tend to indicate decreased muscular activity (which rather stands in contrast to a pronounced increase of T2 relaxation time after initiation of exercise) and with the soleus muscle being a predominantly slow-twitch muscle, the reliability of the demonstrated correlation seems rather questionable. Overall, possible implications should be re-examined in the context of a larger study cohort with consecutive subgroup analyses.

Since many of the overuse injuries mentioned in the introduction are related to muscle dysfunction or fatigue especially of the TA [20], this study could serve as a basis for further studies with the aim to detect and prevent impending overuse injuries at an early stage. To elaborate, our results can be interpreted in combination with the results of Sanno et al [48]

In summary, our results suggest that moderate muscle activity can be better monitored with T2 relaxation time than with T2* relaxation time. Presumably, T2* relaxation time can be helpful in measuring muscular activity during maximum exercise or in the study of muscles with a high proportion of fast-twitch fibres [13]. It can be assumed that other factors, such as the training status, age and BMI have an influence on T2* and T2 relaxation time; however, detailed analyses in this respect were outside the scope of this study.

The limitations of the study are in particular the small number of participants. A subgroup analysis or comparison between slower and faster runners was therefore not possible. Also, with regard to the heterogeneity of recreational activities, possible influences on the results cannot be excluded and a more homogeneous group should be targeted in subsequent studies. A correlation between our results and physiological markers like intramuscular or intravenous lactate concentration would have been helpful to put our results into a physiological context. This would also have been useful to determine a more accurate individual maximum running speed instead of the subjectively chosen running speed, which is also a limitation of our study. A comparison with other quantitative imaging techniques such as diffusion tensor imaging or MR spectroscopy would also have been interesting, however, was beyond the scope of this study, as the pauses between runs would have been too long. Another limitation of our study is that T2* and T2 mapping cannot be performed simultaneously. However, since T2* mapping was always performed first, the observed differences between T2* and T2 relaxation time are not explained by a delay in the T2* relaxation time measurements.

## Conclusion

T2* and T2 relaxation time showed a similar course with an initial rapid increase, a plateau phase (of about 2.5–15 minutes) and a subsequent decrease under prolonged exercise. Moderate but long-term muscular activity appears to have a weaker effect on the T2* relaxation time than on the T2 relaxation time. From this we can conclude that the T2 relaxation time is better suited for monitoring moderate but long-lasting muscle activity.

## Supporting information

S1 Dataset First tab: Volunteer details. Second tab: T2 relaxation times. Third tab: T2* relaxation times.
(XLSX)

S1 Fig Example of muscle segmentation. The borders of the segmented muscles are shown as a green line. The segmentation has been performed in the first echo of the T2-sequence (a), as it provided the best contrast for the anatomical structures, and then copied to the T2 map (b) and the T2* map (c).
(TIF)
