# Supplementary figures and images for: The behaviour of T2* and T2 relaxation time in extrinsic foot muscles under continuous exercise: A prospective analysis during extended running
Source: PLoS One. 2022 Feb 17;17(2):e0264066. doi: 10.1371/journal.pone.0264066 (PMC8893273; doi:10.1371/journal.pone.0264066)

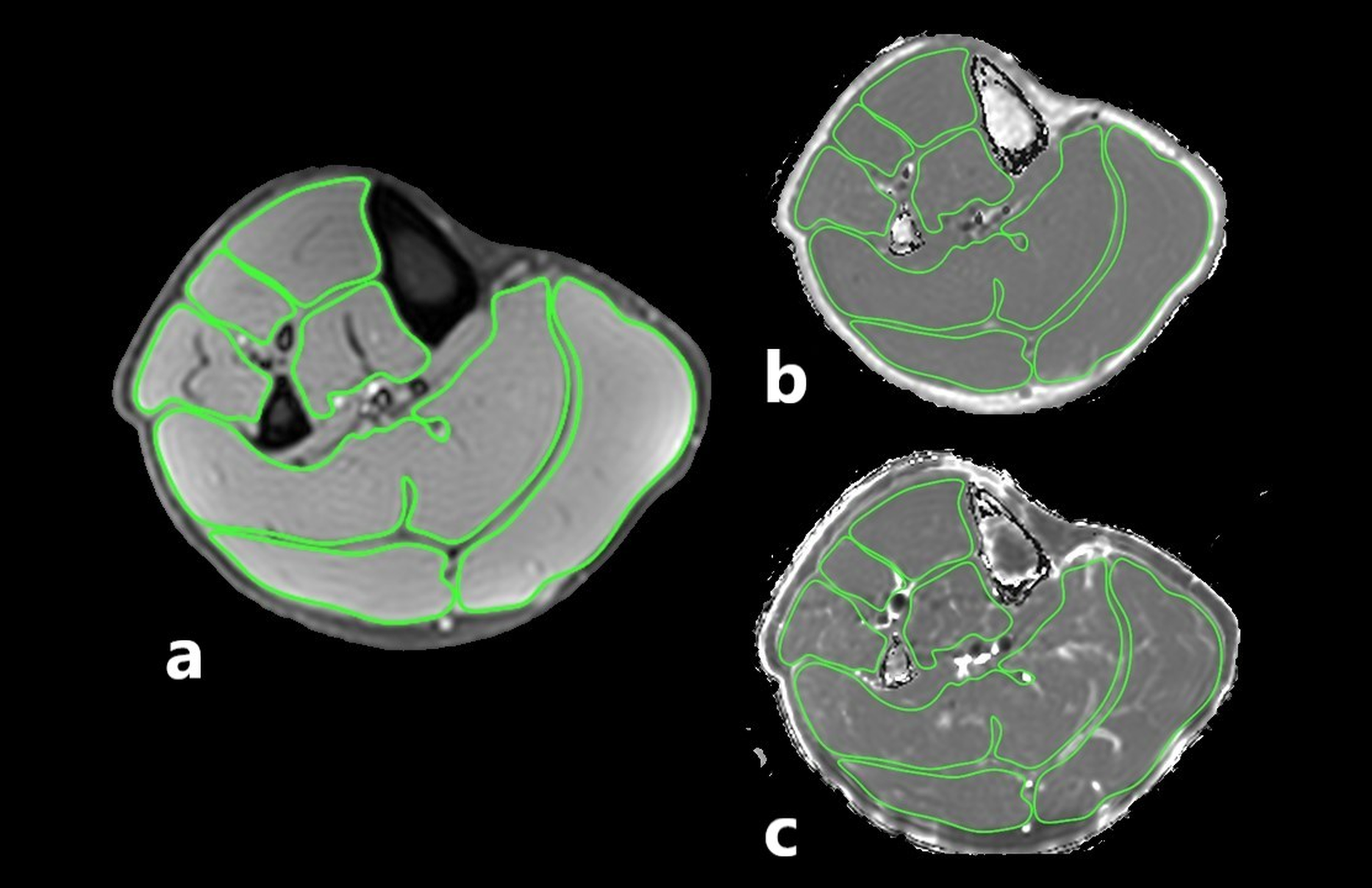

Supplement: S1 Fig — The borders of the segmented muscles are shown as a green line. The segmentation has been performed in the first echo of the T2-sequence (a), as it provided the best contrast for the anatomical structures, and then copied to the T2 map (b) and the T2* map (c). (TIF) [file pone.0264066.s002.tif]
